# 

**Published:** 1893-08-12

**Authors:** 


					? aomfur
Dd.   gffQUST IgA'H, IggJ.
0.359 Vnl vXW?PENCE. II with_e^ra_nurs1ng_3uppleMent.
XIV,--Aug. 12th, 1893. JF5? K* a
tran*mi??ioniiithamii^1v-:?3P? as ft Newipaper for ]??> 0
per Annum, 8s. 6d., post free.
Monthly Parts. 6d.j post free, 8d.
^theUniwii^?nViSSST10' ^ ^ ^ Ditto per Annum} 8s., post free
C,
Ci-ASGOnr ^?srfR^lNFi-RMARy
Nov 359. Vol. XIY. Saturday, August 12th, 1893. [Twopence.
7?? ? WITH EXTRA HURSINfl 8UPPLEMEKT. C>=^-

				

